# Intermittent Fevers

**Published:** 1849-01

**Authors:** 


### Article VI.—*Intermittent Fevers.*

M. Bouchardat conceives that intermittent fevers may be usefully arranged under four forms or varieties, according to the intensity of the disease, and the corresponding effect of quinine on its course and duration. The *first form* includes those obstinate cases which require large doses (15 to 30 grs.) of quinine for their temporary cure, but in which, in spite of the methodical use of the remedy, the fever continues to recur for years. This variety is found in Italy, North of Africa, &c., and is contracted, according to Bourchardat, by a lengthened residence in the neighborhood of marshes, the water of which contains sulpho-salts in solution. To the *second form* belong those cases which require, but may be effectually cured by large doses (15 to 25 grs.) of quinine. An intermittent of this kind prevails at Tours, and has been well described by M. Bretonneau, who observes that small doses are here insufficient. They habituate the patient to the action of quinine, irritate the stomach, and render it difficult to obtain the full action of large doses. The *third form* comprises those mild cases in which small doses (1 to 5 grs.) are sufficient not only to arrest the fever, but also to prevent its return. Lastly, he includes in a *fourth form*, the numerous cases in which change of residence, or admittance into hospital, is sufficient to remove the disease.—*South. Med. Jour.*

